# ZrO_2_ Ceramic without and with Fullerene C_60_ Films: In Vitro Direct-Contact Model Using *E. coli* and *S. aureus* Bacteria

**DOI:** 10.3390/jfb17040206

**Published:** 2026-04-21

**Authors:** Annett Dorner-Reisel, Jialin Li, Marta Trzaskowska, Vladyslav Vivcharenko, Jiacheng Chu, Emma Freiberger, Uwe Ritter, Agata Przekora, Aneta Zima, Tao Wang, Jens Moje

**Affiliations:** 1 Faculty of Mechanical Engineering, Materials Science & Technology, Schmalkalden University of Applied Sciences, 98574 Schmalkalden, Germany; 2 Faculty of Medical Science, Department of Tissue Engineering and Regenerative Medicine, Medical University of Lublin, Chodzki 1, 20-093 Lublin, Poland; 3 Institute for Chemistry & Bioengineering, Technical University of Ilmenau, 98693 Ilmenau, Germany; 4 Faculty of Materials Science and Ceramics, AGH University of Krakow, 30-059 Krakow, Poland; 5 College of Materials Science & Technology, Nanjing University of Aeronautics and Astronautics, Nanjing 210016, China; 6 Moje Keramik-Implantate GmbH & Co. KG, 07616 Petersberg, Germany

**Keywords:** zirconia bioceramic, Gram-positive bacteria, cytotoxicity analysis, Raman spectroscopy, fullerene C_60_, surface pattern

## Abstract

Zirconia is known as a strong and bioinert load-bearing material for dental implants. It typically exhibits no antibacterial activity. Inflammation is a crucial problem for dental implant surgery: about 3–5% of all dental implants experience inflammation. This study demonstrates that either fullerene C_60_ films or a tribomechanical loading of zirconia without the fullerene C_60_ coating can cause an improvement in antibacterial activity against Gram-positive *Staphylococcus aureus*. This moderate antibacterial activity is especially important, because a strong antibacterial effect could disturb the sensitive and beneficial oral bacterial biota. In the present study, different fullerene C_60_ films were examined. In addition to fullerene C_60_ film in an “as deposited” condition, treatment with nitrogen plasma as well as tribomechanical produced surface patterns with and without plasma post-treatment were tested. An 85.8% (log reduction 0.85) reduction in Gram-positive *Staphylococcus aureus* bacterial formation was observed on the zirconia with fullerene C_60_ film. Plasma treatment of the C_60_ film increases the antibacterial impact to 72.2% (log reduction 0.56) in comparison to zirconia without fullerene C_60_ film. Also, tribomechanical loaded fullerene C_60_ films suppress the growth of Gram-positive *Staphylococcus aureus.* The tribomechanical loading seems to compensate for the effect of the plasma treatment. ZrO_2_ samples with fullerene C_60_ film and tribomechanical loading achieve an increase in antibacterial impact of 83.36% (log reduction 0.78). Furthermore, surprisingly yttria-stabilized zirconia bioceramic without fullerene C_60_ film also shows an improved antibacterial efficacy after a tribomechanical patterning procedure. The addition of surface patterning on the ZrO_2_ by scratching microgroove arrangements with a diamond tip, increased the antibacterial effect against Gram-positive *Staphylococcus aureus* by 70.46% (log reduction 0.53).

## 1. Introduction

Zirconia (ZrO_2_) is an important bioinert bioceramic [1]. Bioinert ceramics are chemically stable with no harmful effects when implanted into living tissue, including the provocation of any immune response: bioinert behavior also means that there is no cell growth-promoting effect. On the macro scale, ZrO_2_ bioceramics show little or no antibacterial activity; however, ZrO_2_ nanoparticles have been proven to exert antibacterial action towards both Gram-positive and Gram-negative bacteria [2,3,4].

Good wear resistance, high compressive strength and good fracture toughness, which may be further improved by yttria stabilization, make this ceramic highly suitable for dental implants [5]. Although the mechanical qualities of zirconia dental implants are good, biofilm imbalance and teeth inflammation can lead to serious problems [6]. Oral *Streptococci* are the first microorganisms that colonize oral surfaces. They are considered important microorganisms in the human mouth biota and need to be in balance to prevent pathogen penetration into the human body. Various systemic diseases result from strong bacterial penetration into the human body from the oral cavity, such as infective endocarditis, purulent infections, brain hemorrhage, intestinal inflammation, and autoimmune diseases, as well as bacteremia; all may result from an unbalanced oral biota [6,7].

Unbalanced biofilm formation also contributes to inflammation. In the United States, over 500,000 biofilm-related implant infections occur annually [8] and the unbalanced biofilm is an important cause of lowered wear resistance in zirconia implants. Figueiredo-Pina et al. [5] report the influence of a *Streptococcus salivarius* biofilm on tribomechanical properties. *S. salivarius* is a Gram-positive bacterium that is part of the normal flora of the human mouth. *S. salivarius* plays an essential role in preventing inflammation and tooth decay. It is known for its antibacterial properties, restraining the growth of harmful bacteria such as *Streptococcus mutans*, the primary cause of tooth decay [6,9]. The aim of the study is to gain a first inside about antibacterial effects of fullerene C_60_ films on zirconia in different surface conditions. Both Gram-positive and Gram-negative bacteria are included. At the same time, cytotoxicity should be assessed. The condition of the fullerene C_60_ and the zirconia is monitored by Raman spectroscopy.

## 2. Materials and Methods

### 2.1. Preparation of the Fullerene C_60_ Films

Fullerene C_60_ films were generated on cylindrical medical-grade ZrO_2_ with 3 mol.% yttria at the Institute for Chemistry and Bioengineering, Technical University of Ilmenau, Germany. The samples were 14 mm in diameter and 2 mm thick. The cylinders were provided by Moje Keramik-Implantate GmbH und Co. KG (Petersberg, Germany). The medical-grade surface conditions provided by the company are named “as delivered” in this study. The surface of the bioceramic samples was prepared by pressing ceramic powder in steel dies to a final pressure of 135 MPa. After de-binding and sintering at a temperature of 1250 °C for 2 h, six cylindrical samples were placed in a quartz tube, which was in turn placed in a quartz crucible with the 99% purity fullerene C_60_ powder. The powder was placed in the center of the quartz tube, which was evacuated down to a pressure of 1.4 × 10^−6^ mbar.The quartz crucible was then heated to 540 °C, vacuum sublimate the C_60_ molecules. This sublimation process was continued for 4 h when the quartz tube was allowed to cool for 12 h.

### 2.2. Generation of Surface Patterns by Diamond-Pin Sliding

**Figure 1:**
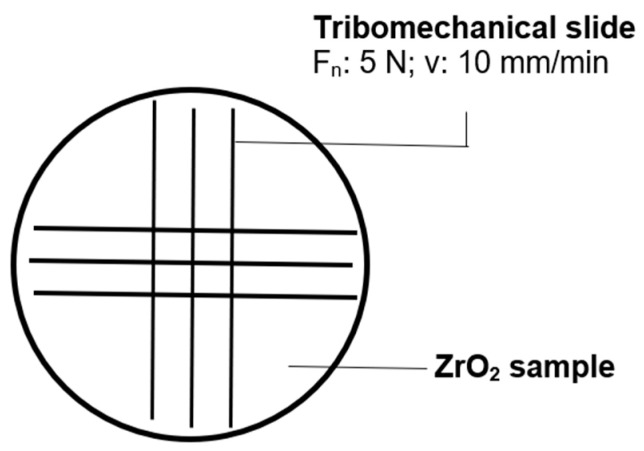
Patterns * for tribomechanical loading of ZrO_2_ surfaces w/o C_60_ and ZrO_2_ + C_60_ surfaces. (*) not true to scale.

Tribomechanical patterning was performed at Faculty of Mechanical Engineering, Materials Science and Technology, Schmalkalden University of Applied Sciences, Germany with an Anton Paar Scratch tester RST 100 (Anton Paar GmbH, Baden, Switzerland). A standardized diamond tip with a tip radius of 0.2 mm and a cone angle of 120° was loaded with 5 N normal force and moved with a velocity of 10 mm/min along the surface of the samples. The tribomechanical pattern is shown in Figure 1.

### 2.3. Plasma Treatment of Samples

The samples were plasma-treated using a “Zepto One” plasma cleaner (Diener GmbH, Ebhausen, Germany). A nitrogen plasma was generated and kept stable with a needle valve, maintaining a gas flow of 0.75 sccm. At 13.56 MHz, the power output is adjustable between 0 and 200 W. A borosilicate glass sphere with a diameter of 105 mm and a volume of approx 2.6 L was used as a vacuum chamber. The electrodes for plasma generation are positioned outside the vacuum chamber. Nitrogen plasma treatment was carried out for 10 min for uncoated ZrO_2_ samples, and the ZrO_2_ + C_60_ samples with and without tribomechanical patterns.

### 2.4. Microscopic Characterization

A Leica DM4 B optical microscope (Leica Microsystems GmbH, Wetzlar, Germany) was used for microscopic characterization at 5×, 20× or 50× magnification. A digital KEYENCE VXH950 microscope was used to create 3-dimensional images of the tribomechanical patterned samples.

### 2.5. Raman Spectroscopic Characterization

Raman spectroscopy was performed at the Faculty of Mechanical Engineering, Materials Science and Technology, Schmalkalden University of Applied Sciences, Germany, using Renishaw inVia QZZ5394 (Renishaw GmbH, Pliezhausen, Deutschland) equipped with a 45 mW monochromatic 532 nm doubled Nd:YAG laser with ~1.0 cm^−1^ spectral resolution. The cylindrical samples were placed on a motorized stage with ±1 µm X-Y repeatability and accuracy. Each measurement was done at a 50× magnification level with an acquisition time of 10 s and five repetitions. Spectral ranges of 100–900 cm^−1^ and 100–3200 cm^−1^ were used to examine peaks of interest. Spectra were analyzed and processed with the Renishaw WiRE 4.4 software. Each spectral array underwent baseline correction to remove the background noise. Peak position, amplitude, and HWHM were collected for different peaks in each spectrum.

### 2.6. Cell Culture Experiments

Cytotoxicity of the tested samples was assessed at the Faculty of Medical Science, Department of Tisuue Engineering and Regenerative Medicine, Medical University of Lublin, Poland using a normal human fetal osteo-blast cell line (hFOB 1.19) obtained from ATCC (American Type Culture Collection, Teddington, UK). Cells were cultured in DMEM/Ham F12 (Sigma-Aldrich Chemicals, Poznań, Poland) without phenol red supplemented with 10 % fetal bovine serum (Pan-Biotech GmbH, Aidenbach, Germany), 100 µg/mL streptomycin, 100 U/mL penicillin (Sigma-Aldrich Chemicals, Poznań, Poland), 2.5 mM L-gluta-mine, and 300 µg/mL G418 at 34 °C in a humidified atmosphere of 5% CO_2_.

#### Cytotoxicity Assessment

The cytotoxicity of the synthesized samples was evaluated at the Faculty of Medical Science, Department of Tissue Engineering and Regenerative Medicine, Medical University of Lublin, Poland, according to ISO 10993-5:2009 (Biological Evaluation of Medical Devices—Part 5: Tests for In Vitro Cytotoxicity. ISO: Geneva, Switzerland, 2009.) and ISO 10993–12:2021 standards (Biological Evaluation of Medical Devices—Part 12: Sample Preparation and Reference Materials. ISO: Geneva, Switzerland, 2021). Briefly, 3 cm^2^ of each tested sample was immersed in 1 mL of DMEM/Ham F12 for 24 h at 37 °C to prepare sample extracts. Simultaneously, 100 µL of hFOB 1.19 cells at a concentration of 1.5 × 10^5^ cells/mL were seeded in flat-bottom 96-well plates and cultured for 24 h. Afterwards, 100 µL of the culture medium in each well was replaced with an equal volume of the prepared extract, and the cells were further cultured for 24 h. Following incubation, cytotoxicity was analyzed using WST-8 and LDH total assays (Sigma-Aldrich Chemicals, Warsaw, Poland) in accordance with manufacturers’ protocols. Additionally, the cytotoxicity was assessed using a Live/Dead Double Staining Kit (Sigma-Aldrich Chemicals, St. Louis, MO, USA) in direct contact with biomaterials. For this purpose, the tested biomaterials were placed in flat-bottom 24-well plates, and 500 µL of hFOB 1.19 cells at a concentration of 1.5 × 10^5^ cells/mL were seeded per material. After 72 h of culture, the hFOB 1.19 cells were stained with calcein-AM and propidium iodide according to the manufacturer’s protocol and visualized using a confocal laser scanning microscope (CLSM, Olympus Fluoview equipped with FV1000, Olympus Corporation, Tokyo, Japan). Biological assays were conducted in 3 independent experiments.

### 2.7. Microbiological Experiment

To assess the antibacterial properties of the biomaterials, the following bacterial strains were employed: Gram-negative *Escherichia coli* ATCC 25922 and Gram-positive *Staphylococcus aureus* ATCC 25923. The strains were initially cultured on Petri dishes containing fresh Mueller–Hinton agar (MHa, Biomaxima, Lublin, Poland) and incubated at 37 °C for 24 h. Subsequently, a single colony from each strain was transferred into Mueller–Hinton broth (MHb, Biomaxima, Lublin, Poland) and incubated under the same conditions. The resulting bacterial suspensions were then appropriately diluted to achieve the required cell density for further analysis. The mean value and the standard deviations were calculated from the results of six samples.

#### Test in Direct Contact with the Material

The antibacterial properties of materials were evaluated by determining the number of viable bacteria following direct contact with the sample surface. The procedure followed the OECD guidelines for non-porous materials (Standard No. 202, JT03360420). Each tested material was evaluated in triplicate, while the control sample (lacking antibacterial activity) was tested in six replicates—three to measure colony-forming units (CFU) immediately after bacterial application, and three after 24 h of incubation. To prepare the bacterial inoculum, a 0.5 McFarland suspension was diluted 1:250 using a 1/50 dilution of MHb in distilled water. This 1/50 dilution was chosen based on experimental results, as the 1/500 dilution recommended by the OECD standard did not sustain sufficient bacterial viability on control samples after 24 h. The final inoculum had a density of 6 × 10^5^ CFU/mL. The inoculum was applied to the surface of each sample and covered with a polyethylene film to ensure uniform contact. Samples were placed in Petri dishes and incubated at 37 °C for 24 h. Half of the control samples were analyzed immediately after inoculation. After incubation, the bacteria were collected by pipetting the sample surfaces multiple times with Eugon LT 100 broth (neutralizer, BTL, Warszawa, Poland). The recovered bacteria were quantified using serial dilutions and the pour plate method. Plates were incubated for 24 h at 37 °C, after which bacterial colonies were counted using a Scan 300 colony counter. Antibacterial effectiveness was expressed as both a percentage and a logarithmic reduction in bacterial counts. Each sample was tested in three independent replicates (n = 3).

## 3. Results

### 3.1. Optical Microscopy

**Figure 2:**
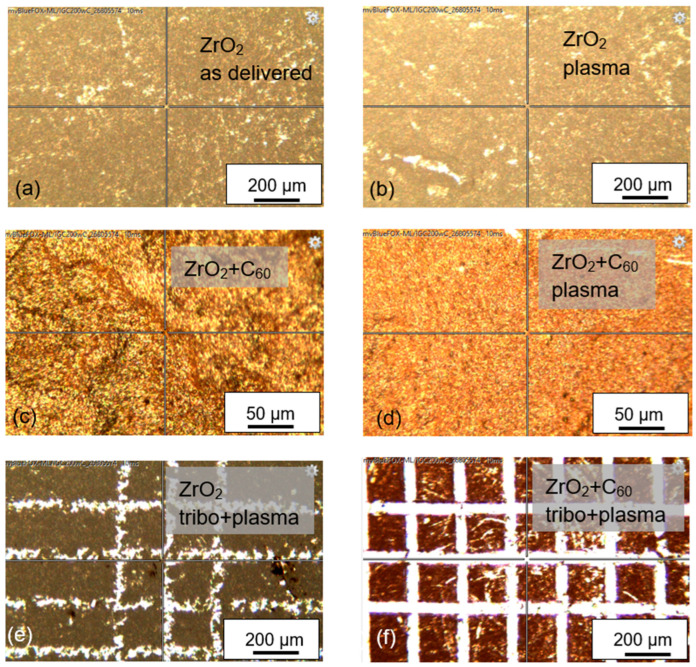
Surface images of several samples: (**a**) ZrO_2_ “as delivered”; (**b**) ZrO_2_ after plasma treatment; (**c**) ZrO_2_ with fullerene C_60_ film; (**d**) ZrO_2_ with C_60_ film after the plasma treatment; (**e**) ZrO_2_ after tribomechanical loading and plasma treatment; (**f**) ZrO_2_ with C_60_ film and after tribomechanical loading.

Using optical microscopy, no difference between zirconia “as delivered” and plasma-treated ZrO_2_ can be seen (Figure 2a,b). Some irregularities at the surface of the ZrO_2_ dental bioceramic give a brighter impression. As described before, the dental bioceramic was used in the “as delivered” condition that is produced by the industrial company. Following deposition of fullerene C_60_ molecules by vacuum sublimation, the surface is covered completely with Buckminster fullerenes (Figure 2c). After plasma treatment, the surface appears more homogeneously. Tribomechanical loading of the uncovered ZrO_2_ surface by the diamond tip with a normal force of 5 N shows bright scratches with irregular edges (Figure 2e). The scratch edges appear smoother for ZrO_2_ with fullerene C_60_ film (Figure 2f).

### 3.2. Raman Spectroscopy

**Figure 3:**
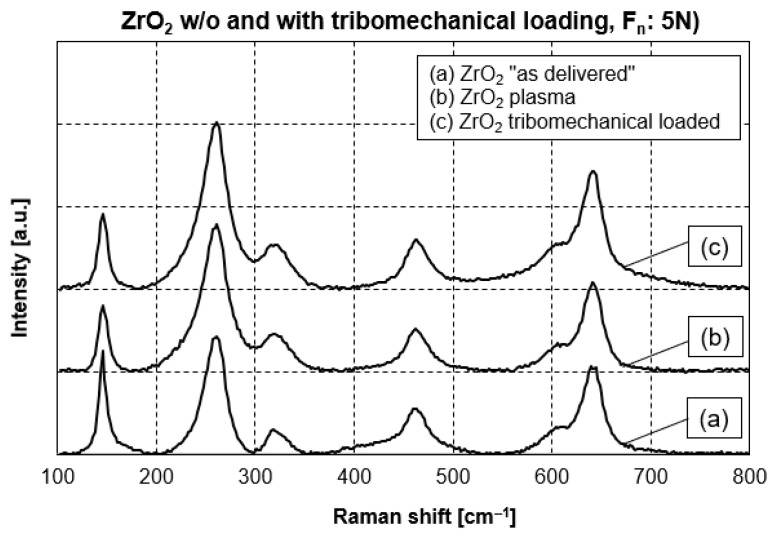
Raman spectra of zirconia without fullerene C_60_ coating.

Zirconia is a polymorph ceramic material appearing in three different crystalline structures: the monoclinic, tetragonal and cubic phases. At room temperature, the monoclinic phase is stable. However, the addition of yttria, in the present study 3 mol-% of yttria is added, does extend the stability of the tetragonal crystalline modification of zirconia to room temperature. The most prominent Raman peaks for each crystalline modification of zirconia according to references [10,11] are summarized in Table 1. In addition, the position of the specific Raman peaks for the different zirconia samples of the present study is included. All samples of zirconia show Raman peaks typical of the tetragonal crystalline modification. The Raman curves in Figure 3 are very similar for the different surface conditions. In comparison to the ZrO_2_ in the “as delivered” state, the plasma treatment, as well as the diamond tip treatment with a normal force of 5 N, did not produce crystalline modifications. In addition, the Raman peaks’ positions do not shift. Therefore, no evidence of any residual stress can be found, even after tribomechanical loading with a normal force of 5 N.

**Figure 4:**
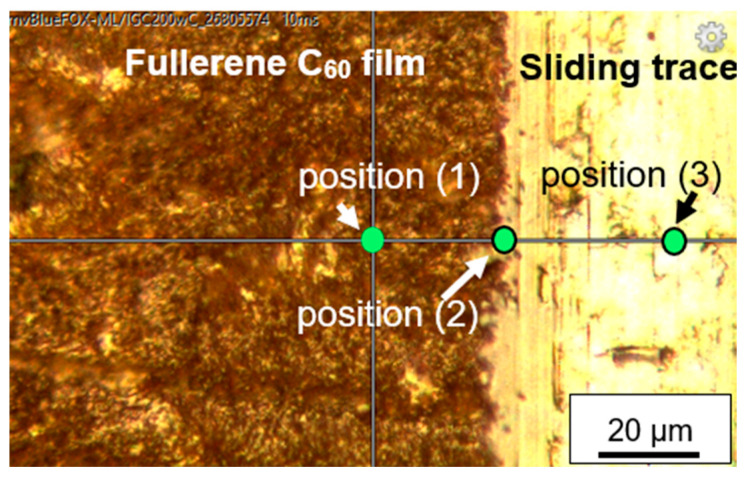
Raman spectra of ZrO_2_+C_60_, tribomechanical patterned.

**Figure 5:**
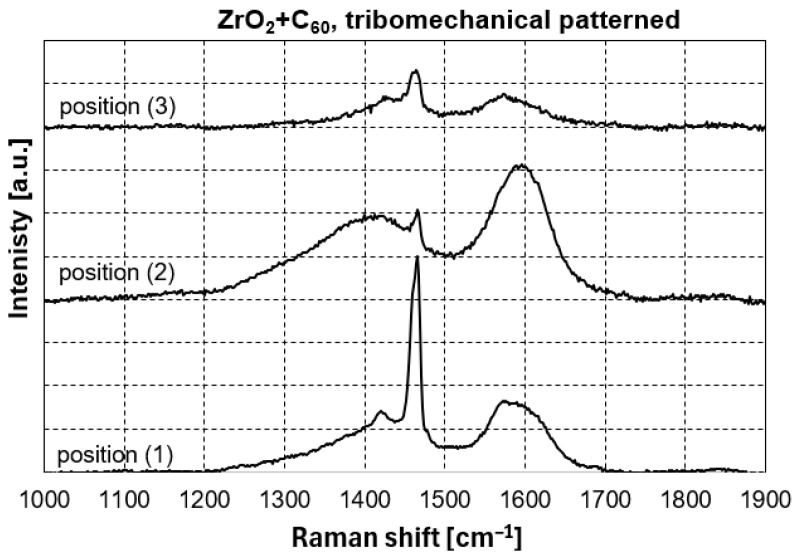
Raman spectra of fullerene C_60_ film on ZrO_2_, position (1): ZrO_2_ + C_60_ without tribomechanical sliding, position (2): ZrO_2_ + C_60_ after tribomechanical sliding *: edge of the scratch, position (3): ZrO_2_ + C_60_ after tribomechanical sliding * with a diamond tip. (*) normal force 5 N; sliding velocity 10 mm/min.

**Figure 6:**
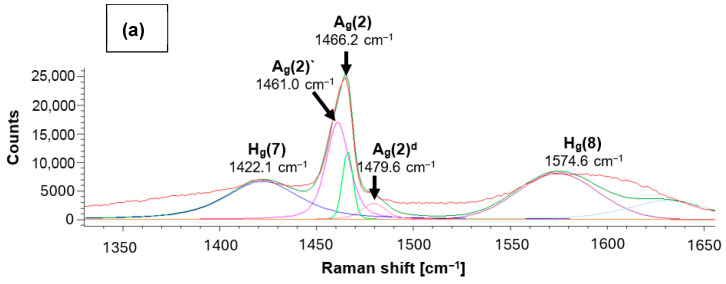

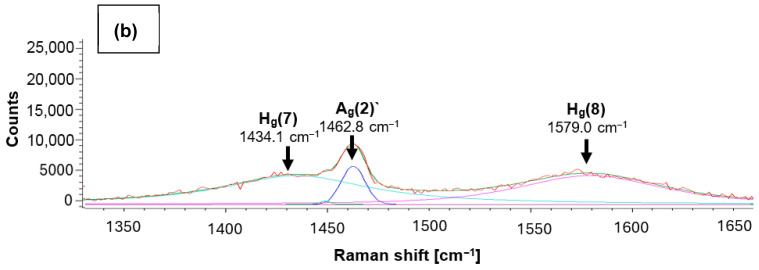
Raman curve deconvolution of fullerene C_60_ film on ZrO_2_: (**a**): position (1): ZrO_2_ + C_60_ without tribomechanical sliding; (**b**): position (3): ZrO_2_ + C_60_ after tribomechanical sliding * with a diamond tip. (*) normal force 5 N; sliding velocity 10 mm/min.

Raman spectroscopy of the fullerene C_60_-coated sample was done on positions with and without tribomechanical loading. Position (1) in Figure 4 was located at the surface of the ZrO_2_ + C_60_ sample without any tribomechanical impact. The prominent region of fullerene C_60_ pentagon pinch mode A_g_(2) can be deconvoluted into 1461.0 cm^−1^, 1466.2 cm^−1^ and 1479.6 cm^−1^ contributions (Table 2, Figure 5 and Figure 6). The Raman signal at around 1461 cm^−1^ can be correlated to dimer formation of fullerene C_60_ buckyballs [12]. The monomeric fullerene C_60_ molecules give a typical Raman signal position around 1466 cm^−1^ if excitation occurred with a green laser source of 532 nm wavelength. It can be seen from Figure 6 that the peak at 1466.2 cm^−1^ is smaller, and the intensity is lower in comparison to the dimer peak at 1461.0 cm^−1^. This points to the fact that the fraction of monomeric C_60_ is lower in comparison to dimers. The Raman signal at 1479.6 cm^−1^ gives evidence of a considerable red shift in the pentagonal pinch mode in fullerene C_60_. It was observed before that monomeric fullerene C_60_ experienced a strong excitation by laser light, which led to the partial deformation of the buckyball cages [12,13]. A substantial red shift in the high-frequency A_g_(2) pentagonal pinch mode was reported by Chase 1992 [14]. These shifts, which increase in the series Au, Cu, and Ag, were in part attributed by Chase et al. to charge transfer to the fullerene by As. Reported previously by us [13], a red shift in the high-frequency A_g_(2) mode to 1479.2 cm^−1^ can occur due to laser irradiation with 22 mW Nd-YAG laser of fullerene C_60_ powder without contact to noble metals. In previous investigations, we reported that Laser irradiation causes C_60_ cage opening and graphene flake generation, accompanied by graphitization with D-peak (disorder peak of sp^2^-hybridized graphite) and G-peak (breath mode of sp^2^-hybridized graphite) formation. In the present study, a partial degradation of fullerene C_60_ may be a reason for the substantial Ag(2) peak red shift to 1479.6 cm^−1^. However, the consensus conclusion on the peak position in the present study and the peak position of former tests at 1479 cm^−1^ points to a specific state of the fullerene C_60_ and not just to the subsequent opening of the C_60_ cage.

At the edge of the scratch, the pentagon pinch mode shifts to 1464.7 cm^−1^. Furthermore, a notable broadening of peaks at 1406.1 cm^−1^ and 1594.7 cm^−1^ can be seen. Rambabu et al. [15] observed a peak broadening for fullerene C_60_ functionalized with sulfonic acid (-SO_3_H) groups. Also, there is a transformation resulting from tribomechanical loading, increasing the pressure and temperature in the micro-areas of the tribological contact between diamond and zirconia with C_60_ film. Raman spectra of fullerene C_60_ show profound changes if sufficiently high pressure and temperatures are reached [16]. There were very broad peaks between 1200 and 1800 cm^−1^ with the highest intensity between 1500 and 1600 cm^−1^, which is typical for highly disordered or amorphous carbon systems [16]. Fullerene-like amorphous carbons contain curved and curled nano-islands of crystalline carbon atoms arranged in hexagonal and/or pentagonal order embedded in amorphous carbon [17,18,19,20].

**Figure 7:**
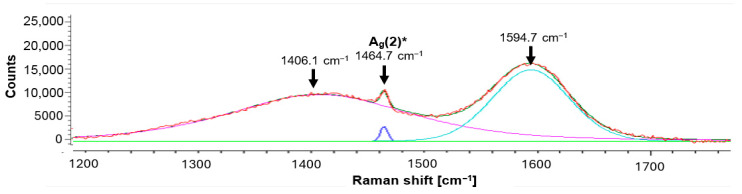
Raman curve deconvolution of fullerene C_60_ film on ZrO_2_: ZrO_2_ + C_60_ after tribomechanical sliding *: edge of the scratch. (*) normal force 5 N; sliding velocity 10 mm/min.

For fivefold rings, the previously mentioned carbon disorder D mode was calculated by Doyle et al. [21] to be located at 1444 cm^−1^, while for six-membered rings, which are the most stable structure, the D band was calculated to be at 1360 cm^−1^, and at 1303 cm^−1^ for seven-membered carbon rings [22]. In the present study, the center of the extremely broad D band is located at 1406.1 cm^−1^. This points to the majority of the disorder carbon rings consisting of fivefold residual rings from the fullerene C_60_ cages. After the transformation of fullerene C_60_ into amorphous carbon, Staresinic et al. [18] found the G-peak position at 1590.0 cm^−1^. The Staresinic et al. finding is also very close to the results of the present study, with a central position of the G peak at 1594.7 cm^−1^ (Figure 7).

Also visible in Figure 7, at the edge of the scratch produced by the diamond tip on top of the ZrO_2_ + C_60_, the residual A_g_(2) Raman peak at 1464.7 cm^−1^ is slightly blue-shifted in comparison to the C_60_ film with no tribomechanical loading. The peak is positioned between the typical dimer C_60_ peak around 1462 cm^−1^ and the monomer peak position around 1466 cm^−1^.

### 3.3. Cytotoxicity Tests

**Figure 8:**
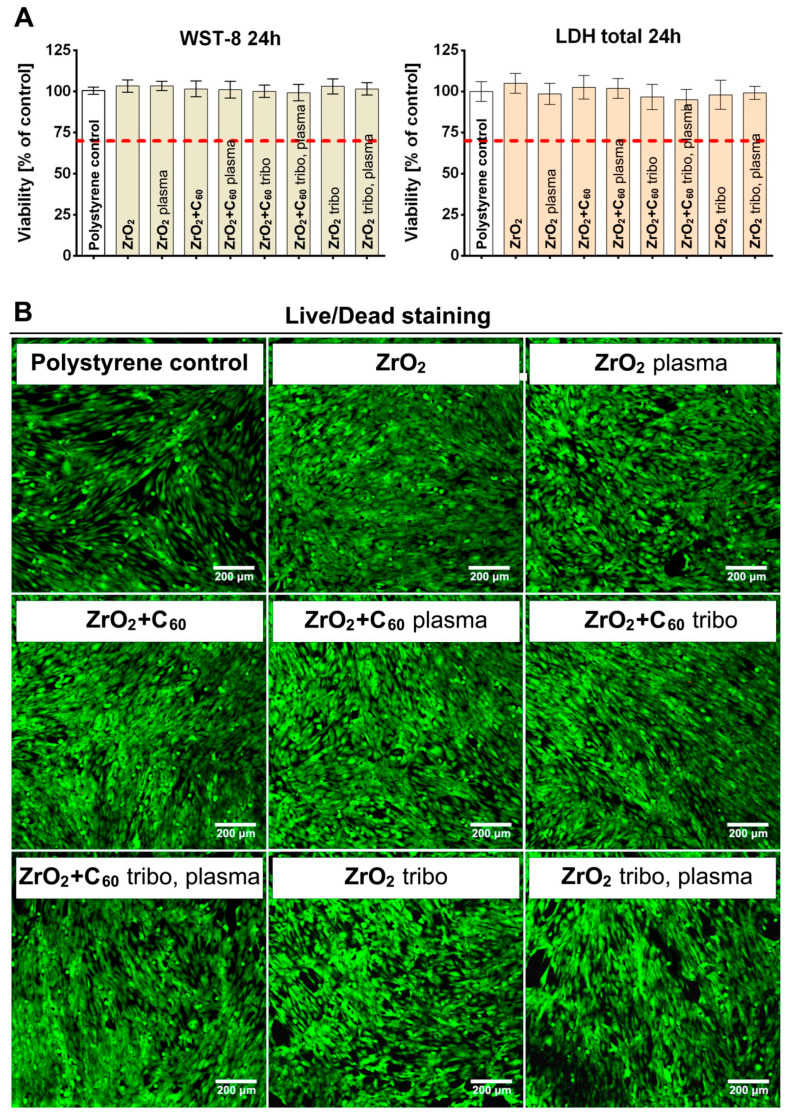
In vitro cytotoxicity evaluation of the synthesized biomaterials against normal human fetal osteoblast cell line: (**A**) WST-8 and LDH total assays performed using biomaterials extracts; the red dashed line represents the ISO 10993-5 cytotoxicity threshold (control—cell treated with polystyrene extract); (**B**) CLSM images of the direct contact cytotoxicity test using the Live/Dead staining kit (red fluorescence—dead cells, green fluorescence—viable cells).

Indirect cytotoxicity tests were performed on the biomaterials in accordance with ISO 10993-5 and showed no cytotoxicity. According to ISO 10993-5, a biomaterial is considered non-toxic if a 100% extract does not cause a reduction in cell viability by more than 30%. Both WST-8 and LDH total assays revealed high cell viability (>95%), indicating the absence of cytotoxic effects on eukaryotic cells for all the modified samples (Figure 8). Due to the bioinert character of the zirconia, the obtained results were, as expected, consistent with those reported in the literature reports [22]. Additionally, fullerene C_60_ films, tribomechanical loading, and plasma treatment of zirconia did not negatively affect cell viability. Comparable results were obtained in the direct cytotoxicity Live/Dead test. CLSM images after 72 h of cell culture on the materials showed that all samples were non-toxic, as no dead cells (red nuclei) were observed. Moreover, the tested biomaterials promoted cell adhesion on their surface, as evidenced by good cell spreading and the normal morphology of osteoblasts. All tested samples were fully covered by the cells, indicating the high biocompatibility of the materials. There was no drop-out.

### 3.4. Microbiological Test

The composition of the resident oral microflora is dominated by bacteria, both Gram-negative and Gram-positive. A stable microflora performs many important functions, including protecting the host against colonization by exogenous populations, which are often pathogenic [23]. Poor oral hygiene, a diet high in sugar or antibiotic therapy may predispose to oral diseases due to the overgrowth of microorganisms that previously constituted a minority of the microflora. Microorganisms in the oral cavity can also cause disease in other parts of the body, acting as opportunistic pathogens. Zirconia is often recommended as an implant material with a wide range of uses in dentistry. Several authors reported that, in comparison to titanium, zirconium exhibited superior resistance to bacterial adhesion and biofilm formation [24].

**Figure 9:**
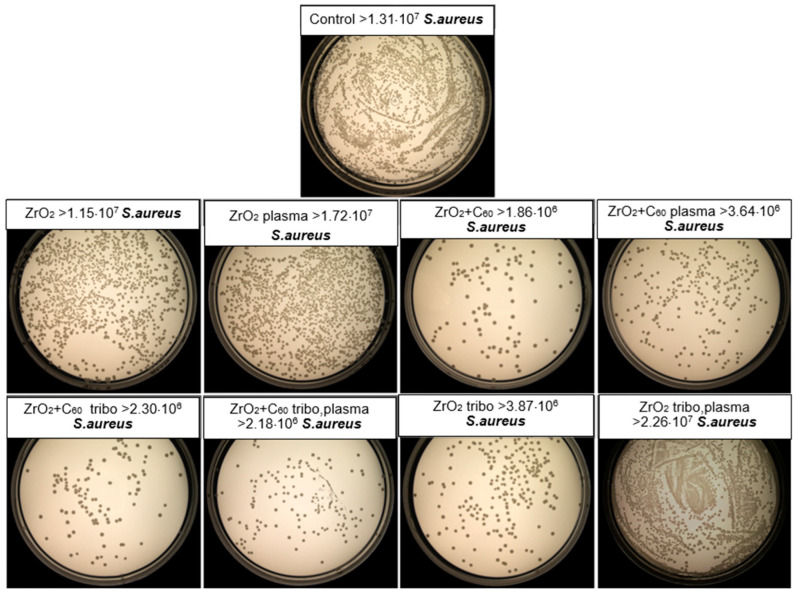
Representative photo of the CFU number of *S. aureus* recovered after 24 h of incubation on the materials’ surfaces, determined by the serial dilution and the pour plate method.

**Figure 10:**
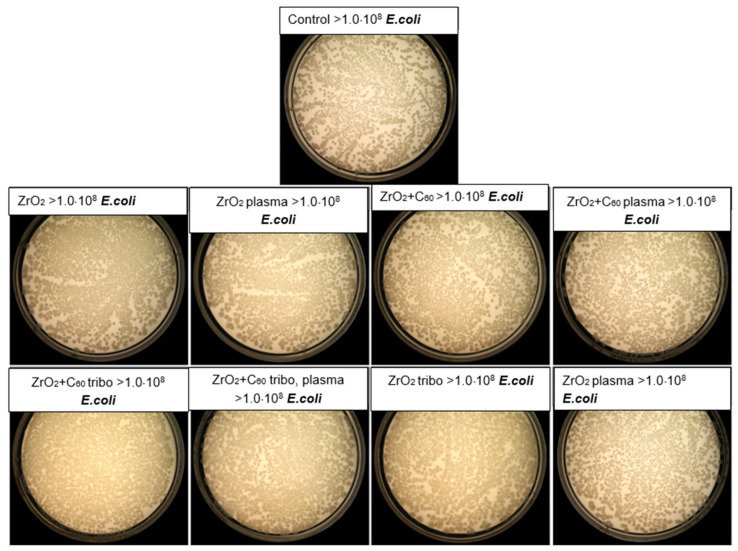
Representative photo of the CFU number of *E. coli* recovered after 24 h of incubation on the materials’ surfaces, determined by the serial dilution and the pour plate method.

The antibacterial activity of the test biomaterials was assessed by direct contact, in accordance with the EOCD standard for non-porous materials. This activity was determined based on the reduction in the number of viable bacteria after incubation on the implant surface with potential antibacterial properties compared to the control material (without any antibacterial activity). The ZrO_2_ + C_60_, ZrO_2_ + C_60_ plasma, ZrO_2_ + C_60_ tribo, ZrO_2_ + C_60_ tribo, plasma and ZrO_2_ + tribo materials showed moderate antibacterial activity with a CFU reduction of 70–86% (log reduction 0.53–0.85) against the Gram-positive bacteria *S. aureus* (Table 3, Figure 9 and Figure 10). There was no drop-out.

ZrO_2_ ceramics demonstrated very slight antibacterial properties, whilst their modification with a tribomechanical pattern increased bacterial CFU reduction by up to 70% (log reduction 0.53). Interestingly, plasma treatment did not affect the antibacterial properties of the implants. No antibacterial activity was observed for any of the tested materials against Gram-negative *E. coli*. The ZrO_2_ + C_60_, ZrO_2_ + C_60_ plasma, ZrO_2_ + C_60_ tribo, and ZrO_2_ + C_60_ tribo plasma materials likely owed their antibacterial properties towards *S. aureus* due to modification with a fullerene C_60_ layer. Fullerenes are characterized by their ability to penetrate microbial cells and then interact with cellular structures. When exposed to light, fullerenes are photosensitizers, producing highly reactive singlet oxygen, the presence of which led to cell death [25]. Research reports indicated that Gram-positive bacteria were susceptible to inactivation using photosensitizers, whereas Gram-negative bacteria exhibited greater resistance, particularly to neutral or anionic photosensitizers. Research results emphasize that photosensitizers were less effective against Gram-negative bacteria due to the presence of a complex outer membrane with a negative surface charge. The outer membrane constituted a barrier that hindered the interaction of photosensitizing compounds with the cytoplasmic membrane, thus blocking the negative effects of reactive oxygen species (ROS) activity [26]. Moreover, it is said that zirconium oxide itself (ZrO_2_) possesses certain antibacterial properties. However, these abilities of zirconia have yet to be thoroughly studied [27,28]. Moderate antimicrobial activity may be beneficial in certain circumstances due to a reduced risk of harming beneficial oral microflora, which could lead to microbial imbalance. It is worth noting that our antibacterial effect was achieved while maintaining a complete lack of cytotoxicity to eukaryotic cells. Whereas highly antibacterial surfaces, especially those containing potent agents, are often cytotoxic to host tissues [29,30].

## 4. Discussion

In the present study, two important mechanisms for the reduction in inflammation due to inappropriate bacterial expansion are described.

**Figure 11:**
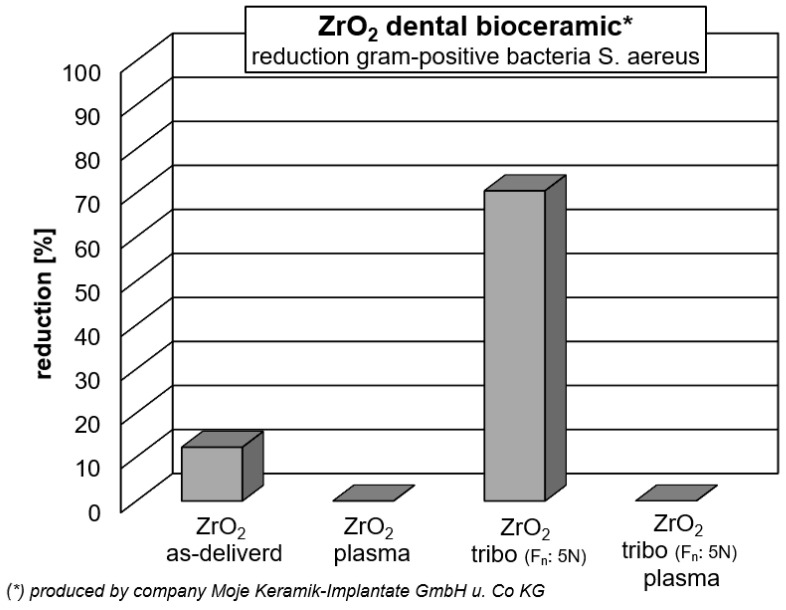
Reduction in Gram-positive *S.aureus* bacteria on medical * grade ZrO_2_. (*) ZrO_2_ with 3 mol.% yttria.

After tribomechanical loading with 5 N, it was found that zirconia has strong antiseptic behavior. In comparison to the polystyrene control, there is a reduction in Gram-positive *Staphylococcus aureus* bacteria of 70.46% (Table 3, Figure 9 and Figure 11). As discussed before [3], zirconia nanoparticles are found to be antiseptic. Nano zirconia ZrO_2_ shows biologically active effects primarily due to its unique nanoscale physicochemical properties. As shown, in the actual study, the bulk zirconia in the “as delivered” condition without coating or tribomechanical loading gave an increased bacterial activity of 12.21%. When ZrO_2_ particles are nanosized, surface area is increased, surface chemistry is altered, enhancing reactivity compared to bulk equivalents. Zirconia can release Zr^4+^ ions into the surrounding liquid, leaving back ion vacancies and negatively charged oxygen in the lattice, which results in a negatively charged defective zirconia nanoparticle [31,32,33]. Lv et al. [32] describe the electrostatic adsorption of positively charged ions onto defective zirconia nanoparticles. In the present study, due to the 5 N normal force tribomechanical loading, the generation of wear debris and particles, including nanoparticles of the ceramic zirconia, is expected. Such tribomechanically induced nanoparticles may be one possible reason for the strong increase in antibacterial activity after the sliding diamond tip treatment. If one considers that there was just a small section of the surface of zirconia scratched by the diamond tip, more and stronger antibacterial activity can be expected if the whole surface is properly tribomechanically loaded and stimulated due to restricted surface cracking and nanoparticle formation from the inherently brittle ceramic material.

Furthermore, nano-scale zirconia shows enhanced piezoelectricity related to size [34,35,36,37]. Roy et al. [35] generated ZrO_2_ films with 10 nm, 20 nm and 40 nm thickness by pulsed laser deposition. The zirconia films with 10 nm and 20 nm thickness retain high out-of-plane residual strain. Roy et al. confirm the tetragonal phase of zirconia without any doping but residual strain due to compression of the energetic ion bombardment during the ZrO_2_ film generation via pulsed laser deposition. The 40 nm thick ZrO_2_ film exhibited relaxation phase transition into monoclinic structure without any piezoelectricity and polarization. In the present study, zirconia in the tetragonal phase was determined by Raman spectroscopy in the “as delivered” condition as well as after tribomechanical loading with 5 N (Figure 3). It needs to be taken into consideration that the medical-grade zirconia used in the present study was stabilized by 3 mol.% yttria (see Section 2.1 Materials). Clearly, the tetragonal structure was maintained during the stressing from tribomechanical loading.

So, there are at least two reasons for antibacterial activities of the medical grade zirconia-based bioceramic: (1) the piezoelectricity of loaded surface regions; (2) the generation of nano-zirconia particles with increased surface activity and Zr^4+^ ion release. Considering reason (1), the stresses during the tribomechanical loading may favor the piezoelectricity and lead to increased electric charging effects and interaction with bacteria. At that stage, the extent of generating wear debris and its size distribution needs further research.

However, in the present study, no antibacterial activity of zirconia against Gram-negative bacteria, *Escherichia coli*, was measured (Table 3, Figure 10). It is known that the charge and chemical moieties of the outer membrane of Gram-positive bacteria (i.e., *S. aureus*) and Gram-negative bacteria (i.e., *E. coli*) differ significantly. Sant et al. [37] present results that showed ZrO_2_ nanoparticles’ antibacterial activity only against Gram-negative *E. coli* bacteria and no activity against *S.aureus*. This finding is quite the opposite of the results of the present study. Here, there was no antibacterial activity against Gram-negative bacteria, *E. coli,* measured (Table 3, Figure 10). Sant et al. argue that not only does the size of the ZrO_2_ nanoparticles, but also their different shapes, i.e., different active facets, influence the antimicrobial activity.

Chemically modified Zr^4+^ ions with different amino acids as ligands were synthesized by Sant et al. [37]. Their Zr^4+^ ions with amino acid ligands exhibited antibacterial activity against both Gram-negative and Gram-positive bacteria. Amino acids contain an amino group (-NH_2_) and a carboxyl group (-COOH). Amino acids differ essentially in structure, chirality, polarity or side chains. A simple generation of functional groups by bombardment with nitrogen ions in a nitrogen plasma (see point Section 2.3 Plasma treatment of samples), as done in the actual study, is obviously not sufficient for the generation of antibacterial activities against Gram-positive *S.aureus* or Gram-negative *E. coli* bacteria (Table 3, Figure 9 and Figure 10). The plasma treatment of medical-grade ZrO_2_ and of ZrO_2_ + C_60_ did not result in any antibacterial effect. Plasma treatment substantially modified the wetting behavior of C_60_, resulting in a well-defined super-hydrophilic surface state (see Figure S1 and Table S1 in the Supplementary Information).

Fullerene C_60_ is hydrophobic and a strong electron acceptor. The interaction with negatively charged electron-donating agents can be expected to be high. One of the simplest explanations of the reduction in bacterial activity may be the simple repulsion due to equal electric loading of electron-attracting fullerene C_60_ cages against the negatively charged agents.The interaction of charged surfaces and particles with cells is considered to be essential [38,39,40].

**Figure 12:**
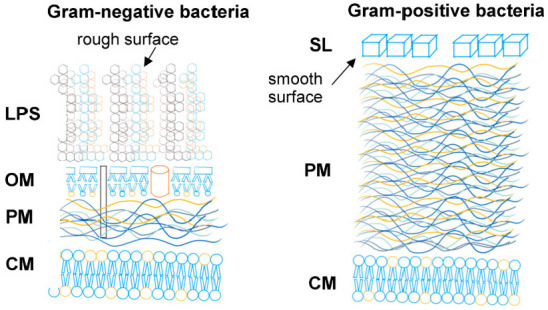
Cell envelope of Gram-negative and Gram-positive bacteria. LPS: Lipopolysaccarchide hair; OM: outer membrane; PM: peptidoglycan; CM: cytoplasmatic membrane; SL: surface layer of crystallized proteins.

All ZrO_2_ surface conditions with the presence of fullerene C_60_ did show a clear increase in antibacterial activity against Gram-positive *S.aureus* bacteria, but not against Gram-negative *E. coli.* Both Gram-positive and Gram-negative bacteria possess a negative surface charge. However, one can expect that the intensity of negative surface charge differs between *S.aureus* and *E. coli* bacteria. Also, every bacterial cell surface adsorbs and desorbs ions and molecules from its surrounding solution; thus, its surface charge characteristics are dependent on its solution milieu. The net surface charge of bacteria can be determined by measuring their electrophoretic mobility or ion adsorption [41]. In addition to a completely different build-up of the outer membrane of Gram-negative and Gram-positive bacteria (Figure 12), the level of negative surface charge certainly influences the interaction with charged surfaces and interfaces, i.e., ion-deficient zirconia or electron-accepting fullerene C_60_. In addition, C_60_ fullerene promotes surficial interactions through the π-π-orbital overlapping [42,43].

**Figure 13:**
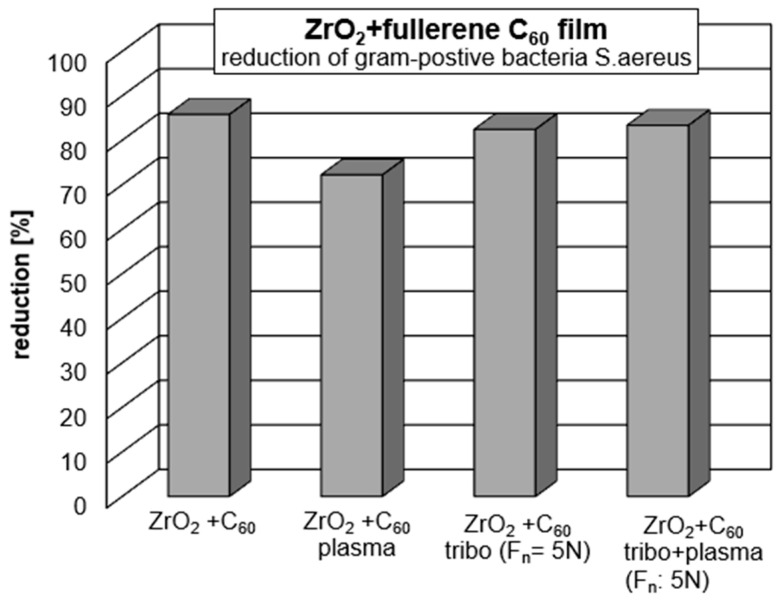
Reduction in Gram-positive *S.aureus* bacteria on fullerene C_60_-coated ZrO_2_ *. (*) ZrO_2_ with 3 mol.% yttria.

In the present study, no cytotoxic effect from any of the C_60_-coated samples was observed. On the contrary, the total assays revealed high cell viability (>95%) (Figure 8). Fullerene in an unexcited state does gently stimulate cell growth and even stem cell differentiation, most probably due to electric charge interactions [44]. In the present study, it was shown by Raman spectroscopy that the fullerene C_60_ coating mainly consists of monomeric molecules (Figure 5 and Figure 6, Table 2). The antibacterial activity is highest for the “as deposited” mainly monomeric fullerene C_60_ film. Plasma treatment leads to the adsorption of molecules at the surface that may shield the fullerene C_60_ surface, increase the distance between bacteria, and reduce electrical interaction with the Buckminster cages. Covering with functional groups reduces the time for the tribomechanically loaded ZrO_2_ + C_60_ samples. Therefore, their antibacterial effect against Gram-positive *S.aureus* increases (Figure 13).

The Fullerene molecules not only absorb UV light but also moderate visible light [45,46]. Light adsorption causes an excited state. Immersed in water, C_60_ shows the highest absorption in the ultraviolet (265 nm, 345 nm) range; two lesser peaks are in blue (450 nm) and red (600 nm) regions of visible light [46].

It is known that another very relevant feature of C_60_ is the ability to quench various free radicals, acting as a ‘‘free radical consumer’’ [47,48,49]. Photosensitization of C_60_ leads to its transition to a long-lived triplet excited state and the subsequent energy or electron transfer to molecular oxygen, yielding highly reactive singlet oxygen (^1^O_2_) or superoxide anion (O_2_). These reactive oxygen species (ROS) react with a wide range of biological targets. They cause oxidative stress and disturb bacteria and cells. This excited state can be reached by UV radiation, while C_60_ adsorbs less in the wavelength range of visible light. As there was no cell damage in all of our cell compatibility studies in the “as deposited” state of fullerene C_60_, we can conclude that the excitation dosage by normal daylight does stimulate the molecules to a limited extent: this stimulation may be sufficient for moderate antibacterial effects. In our study, this reduced the amount of Gram-negative *S.aureus*.

Biofilms are complex and highly dynamic communities [50]. During inflammation, biofilm composition changes. Biofilm population balance is driven by various interactions among its microbial populations, as well as by external parameters such as temperature, pH-value and chemical conditions [51,52]. Interaction between species involves metabolic, physicochemical, regulatory and/or signaling exchanges, which could drive temporary changes in biofilm community function and composition, as well as spatial or general arrangement. Negative stimulation could arise from competition for resources [52], including electron acceptors and donors, light nutrients or spatial structure [49,53,54,55]. Mobelli et al. [56] conducted early studies proving that the microbiome of healthy peri-implant sites mainly consists of Gram-positive cocci, non-motile bacilli, and a few Gram-negative anaerobic species. Peri-implantitis favors anaerobic bacteria over aerobic species due to low oxygen conditions Gram-negative anaerobic bacteria commonly dominate periodontal pockets [56,57]. Such anaerobic conditions should be prevented during implantation and addressed by countermeasures after implantation. The delivery of oxygen at the implant-tissue interface to the fullerene C_60_, for example, by sustainable materials such as glycerol [58,59] could support the moderate but enduring antibacterial effect and extend its efficiency to Gram-negative bacteria by preventing anaerobic conditions. Such aspects need to be proven in future investigations.

## 5. Conclusions and Perspectives

Zirconia surfaces can be tailored to reach an optimal antibacterial effect against Gram-positive and Gram-negative bacteria by understanding and tailoring their surface topography. Nano-scale zirconia particles with suitable shapes and crystallinity can interact with bacteria in different ways.

Fullerene C_60_ films deposited on medical-grade zirconia need to provide close contact with the bacterial surface. The adsorption of other molecules, functional groups, changes the electric charge and electron transfer and reduces interactions with bacteria.

The ability of ion and molecule absorption is dependent on the liquid environment, bacterial membrane structure, charge level and bacteria outer membrane topography and roughness.

The fullerene C_60_ coatings in the mainly monomeric state do not show any harmful effect on cells after moderate illumination by visible light.

In the present study, we just tested single Gram-negative and Gram-positive bacteria without considering the complexity of biofilm community dynamics in the healthy or peri-implantitis situation. We are aware that single-species biofilms are uncommon. In future work, the interaction and coexistence of different typical microbial biofilm compositions need to be quantified.

The first results of the present study confirm a moderate antibacterial activity against Gram-positive *S.aureus* of fullerene C_60_-coated ZrO_2_, as well as tribomechanically loaded ZrO_2_ without fullerene C_60_. No antibacterial efficacy was found against Gram-negative *E. coli* bacteria. However, it dominates peri-implantitis inflammation under anaerobic conditions. Fullerene C_60_ coatings can be further excited and produce reactive oxygen species. One possibility is that fullerene C_60_ could be irradiated immediately after the implantation of biomaterials to reach an initial increased antiseptic effect, followed by support of cell growth without further excitement.

On the other hand, it is conceivable that any peri-implantitis can be much more effectively treated if the affected patient flushes the fullerene C_60_-coated implant with oxygen-containing agents to stimulate strong, temporary in situ reactive oxygen production. Another approach may be the deposition of hybrid films of oxygenating agents with fullerene C_60_.

## 6. Limitation Paragraph

We explicitly state that simplified in vitro model testing was performed in the present study. The study was restricted to single-bacterial assays and did not incorporate a multispecies biofilm model. For future investigations, it is critical to account for biofilm complexity when evaluating the effects of either fullerene C_60_ molecules or tribomechanical loading of zirconia on microbiome composition and biofilm homeostasis. Furthermore, compliance with European Regulations 2017/745 (Medical Device Regulation, MDR) and 2017/746 (In Vitro Diagnostic Device Regulation, IVDR) is mandatory for all medical devices when first placed on the market (i.e., initial supply from the manufacturer to distributors or end users) or put into service (i.e., made available for clinical use) within the European Economic Area (EEA).

## Figures and Tables

**Table 1:** Raman peaks of tetragonal, monoclinic, and cubic polymorphs of ZrO_2_ in the “as delivered“ condition and after different plasma treatment or tribomechanical loading by sliding of a diamond tip with 5 N normal force.

Crystalline Structure of ZrO_2_	Reference Peaks	Raman Peak Position		
	[9,10,11]	ZrO_2_ “As Delivered”	ZrO_2_ Plasma	ZrO_2_ Tribomechanical Loaded
Tetragonal structure	~142 cm^−1^	145 cm^−1^	146 cm^−1^	146 cm^−1^
	~256 cm^−1^	259 cm^−1^	259 cm^−1^	259 cm^−1^
	~320 cm^−1^	322 cm^−1^	322 cm^−1^	322 cm^−1^
	~466 cm^−1^	462 cm^−1^	463 cm^−1^	463 cm^−1^
	~63 cm^−1^	606 cm^−1^	607 cm^−1^	610 cm^−1^
		641 cm^−1^	641 cm^−1^	641 cm^−1^
Monoclinic structure	~178 cm^−1^			
	~190 cm^−1^	-	-	-
	~219 cm^−1^	-	-	-
	~303 cm^−1^	-	-	-
	~331 cm^−1^	-	-	-
	~345 cm^−1^	-	-	-
	~379 cm^−1^	-	-	-
	~474 cm^−1^	-	-	-
	~500 cm^−1^	-	-	-
	~534 cm^−1^	-	-	-
	~559 cm^−1^	-	-	-
	~615 cm^−1^	-	-	-
	~638 cm^−1^	-	-	-
Cubic structure	~628 cm^−1^			

**Table 2:** Raman results: ZrO_2_ + C_60_ with tribomechanical pattern.

Measurement Position	H_g_(7)	A_g_(2)	H_g_(8)
Position (1)	1422.1 cm^−1^	1461.0 cm^−1^ 1466.2 cm^−1^ 1479.6 cm^−1^	1574.6 cm^−1^
Position (2)	1406.1 cm^−1^	1464.7 cm^−1^	1594.7 cm^−1^
Position (3)	1434.1 cm^−1^	1462.8 cm^−1^	1579 cm^−1^

**Table 3:** Assessment of antibacterial activity in direct contact according to the OECD standard.

Bacteria Strain	Mean of the Number of Viable Bacteria (CFU/Sample)				Antibacterial Activity	
	Control Material t = 0	Control Material t = 24 h	Designation of Tested Materials	Tested Materials	% CFU Reduction	Log CFU Reduction
*S. aureus*	3.43 × 10^4^	1.31 × 10^7^	ZrO_2_	1.15 × 10^7^	12.21	0.06
			ZrO_2_ plasma	1.72 × 10^7^	-	-
			ZrO_2_ + C_60_	1.86 × 10^6^	85.80	0.85
			ZrO_2_ + C_60_ plasma	3.64 × 10^6^	72.21	0.56
			ZrO_2_ + C_60_ tribo	2.3 × 10^6^	82.44	0.76
			ZrO_2_ + C_60_ tribo, plasma	2.18 × 10^6^	83.36	0.78
			ZrO_2_ tribo	3.87 × 10^6^	70.46	0.53
			ZrO_2_ tribo, plasm	2.26 × 10^7^	-	-
*E. coli*	1.63 × 10^5^	>1 × 10^8^ (number of bacteria above detection)	ZrO_2_	>1 × 10^8^	-	-
			ZrO_2_ plasma	>1 × 10^8^	-	-
			ZrO_2_ + C_60_	>1 × 10^8^	-	-
			ZrO_2_ + C_60_ plasma	>1 × 10^8^	-	-
			ZrO_2_ + C_60_ tribo	>1 × 10^8^	-	-
			ZrO_2_ + C_60_ tribo, plasma	>1 × 10^8^	-	-
			ZrO_2_ tribo	>1 × 10^8^	-	-
			ZrO_2_ plasma	>1 × 10^8^	-	-

## Data Availability

The original contributions presented in this study are included in the article/Supplementary Material. Further inquiries can be directed to the corresponding author.

## Supplementary Materials

The following supporting information can be downloaded at: https://www.mdpi.com/article/10.3390/jfb17040206/s1, Figure S1: Distilled water droplet on fullerene C60 coated zirconia ZrO_2_ + C_60_ surface in the (a) “as deposited” condition; (b) immediately after plasma treatment; (c) 12 weeks after plasma treatment; Table S1: Wetting angle ∅ for ZrO_2_ and ZrO_2_ + C_60_ before and after plasma treatment.

## Abbreviations

The following abbreviations are used in this manuscript:

C_60_	Fullerene molecule with 60 carbon atoms
ZrO_2_	Zirconia
