# Supplementary material for: ZrO2 Ceramic without and with Fullerene C60 Films: In Vitro Direct-Contact Model Using E. coli and S. aureus Bacteria
Source: J Funct Biomater. 2026 Apr 21;17(4):206. doi: 10.3390/jfb17040206 (PMC13117599; doi:10.3390/jfb17040206)
Supplement: Supplementary file 1 [file jfb-17-00206-s001.zip › jfb-4148706-supplementary.pdf]

Supplementary Materials

S.1 Wetting behavior

The wetting behavior was tested with distilled water. The water droplets were supplied to the zirconia ceramic and ZrO_2+C_{60} samples using a pipette [49]. Ten droplets per sample were generated, measured before calculating the mean value and the standard deviations.

It can be seen from the sessile drop photographs that both materials medical grade zirconia ceramic and fullerene C_{60} modified surfaces ZrO_2+C_{60} showed almost similar wetting behavior. Zirconia had a wetting angle of 75.9 ±2.4 deg while ZrO_2+C_{60} (Figure S1) showed a wetting angle of 71.9 ±2.1 deg (Table S1).

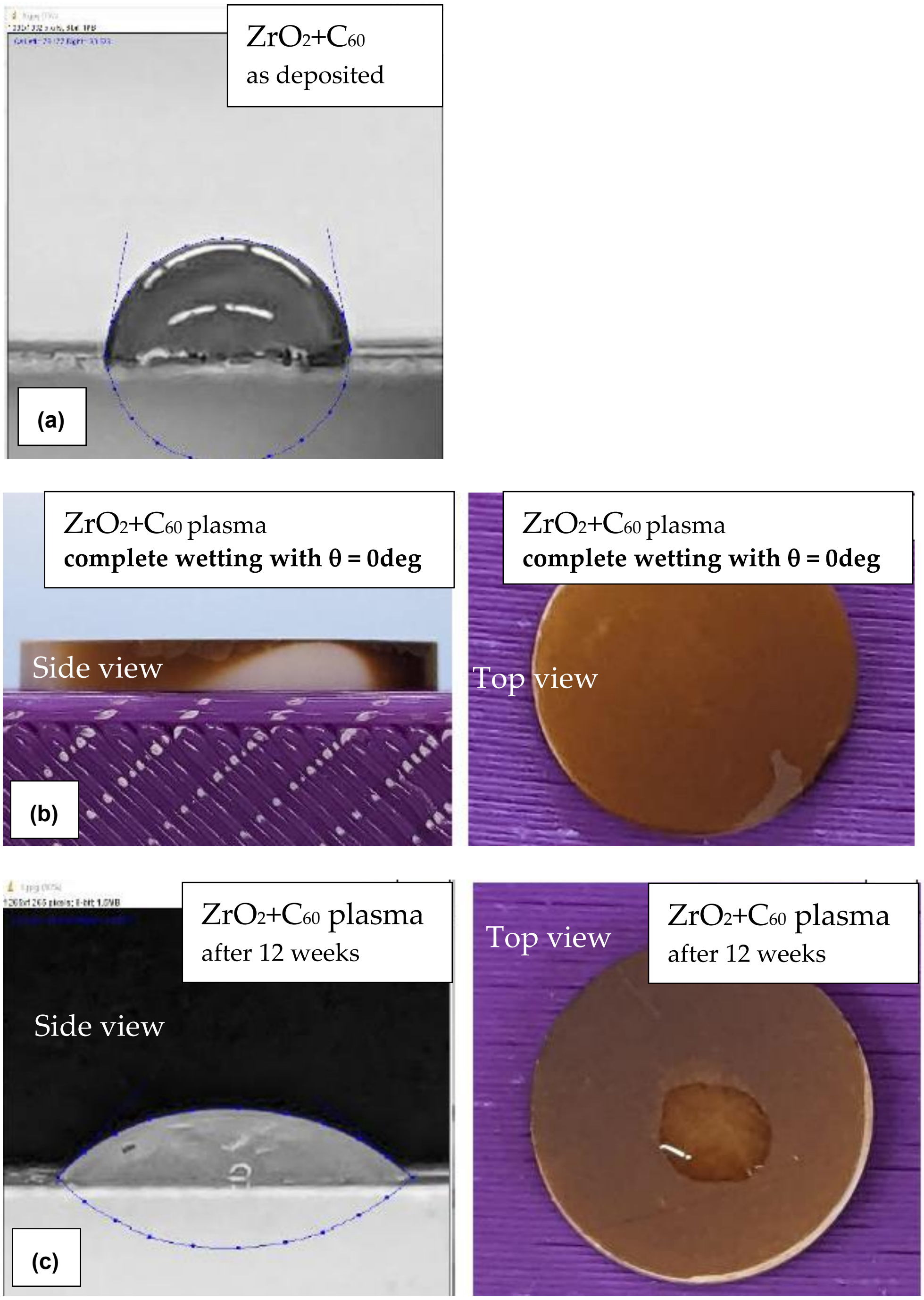

Figure S1. Distilled water droplet on fullerene C_{60} coated zirconia ZrO_2+C_{60} surface in the (a) "as deposited" condition; (b) immediately after plasma treatment; (c) 12 weeks after plasma treatment.

Table S1. Wetting angle q for ZrO_2 and ZrO_2+C_{60} before and after plasma treatment.

Sample	q [deg] before plasma	q [deg] immediately after plasma	q [deg] 12 weeks later
ZrO_2	75.9 ±2.4	-	77.7 ±1.2
ZrO_2+C_{60}	71.9 ±2.1	-	-
ZrO_2+C_{60} plasma*	-	0	28.0 ±0.8

**) plasma treatment was performed 13 min for these tests [49]*
